# Assessment of the Pathogenicity of *Candidatus* Rickettsia Colombiensis in a Syrian Hamster Model and Serological Cross-Reactivity Between Spotted Fever Rickettsia Species

**DOI:** 10.3390/pathogens15020146

**Published:** 2026-01-29

**Authors:** Jorge Miranda, Alejandra García, Cristina Cervera-Acevedo, Sonia Santibañez, Aránzazu Portillo, José A. Oteo, Salim Mattar

**Affiliations:** 1 Instituto de Investigaciones Biológicas del Trópico, Universidad de Córdoba, Córdoba 230002, Colombia; jluismiranda@correo.unicordoba.edu.co (J.M.); agarciaperez@correo.unicordoba.edu.co (A.G.); 2 Centre of Rickettsiosis and Arthropod-Borne Diseases (CRETAV), Department of Infectious Diseases, San Pedro University Hospital—Centre for Biomedical Research (CIBIR), La Rioja, 26006 Logroño, Spain; ccervera@riojasalud.es (C.C.-A.); ssantibanez@riojasalud.es (S.S.); aportillo@riojasalud.es (A.P.); jaoteo@riojasalud.es (J.A.O.)

**Keywords:** pathogenic, cross-reactivity, *Rickettsia*, indirect fluorescent antibody technique

## Abstract

*Candidatus* Rickettsia colombiensis is a new candidate species of Rickettsiae spotted fever group that have been isolated only from ticks. The pathogenicity of *Ca*. R. colombiensis to human and animals is unknown. This study evaluated the pathogenic potential of *Ca*. R. colombiensis in Syrian hamsters and assessed the cross-reactivity between *Ca*. R. colombiensis and other Rickettsia in human and hamster sera. Shell vial technique was employed to isolate *Ca*. R. colombiensis. Subsequently, five male Syrian hamsters were inoculated intraperitoneally (IP) and five intradermally (ID) with 1 × 10^6^ Vero cells infected with *Ca*. R. colombiensis. One control hamster was used in each group. The health status was assessed daily, and necropsies were performed. Serum samples were tested by indirect immunofluorescence and tissues were processed by qPCR and histological stains. All Syrian hamsters remained healthy during the trial. No histopathological damages associated with rickettsial infection were observed. No Rickettsial DNA was detected in tissues. Syrian hamsters showed IgG antibody titers ranging from 1:64 to 1:1024. Control hamsters were negative. Regarding human sera, 56% (84/150) had IgG cross-reactivity antibodies against *Ca*. R. colombiensis. Subsequently, in a selected subset of 30 sera with moderate to high titers, all samples reacted with *Ca*. R. colombiensis antigen. Under specific conditions of this study, *Ca*. R. colombiensis did not behave as a highly virulent pathogen in the hamster model, although all infected Syrian hamsters developed IgG antibodies responses. Regarding cross-reactivity, it is possible to serologically diagnose rickettsial infection using *Ca*. R. colombiensis as an antigen.

## 1. Introduction

*Rickettsiae* are obligate intracellular Gram-negative bacteria of the class of Alphaproteobacteria and are vector-borne species that cause diseases in humans and animals. They are globally distributed and pose a significant public health concern [1]. Pathogenic *Rickettsiae* are associated with hematophagous arthropods, such as ticks, mites, fleas, and lice, which act as transmission vectors and reservoirs in their natural life cycles. However, *Rickettsiae* have also been found in various hosts, including herbivorous arthropods, leeches, and amoebae [2,3].

Based on phylogeny, clinical symptoms, and antigenic properties, *Rickettsiae* are classified into the following three pathogenic groups: spotted fever group (SFG), typhus group (TG), and transitional group (TRG). In addition, a group of non-pathogenic species is poorly studied compared to the pathogenic *Rickettsiae* groups, called the ancestral group (AG) [4,5]. TG comprises two species, *Rickettsia prowazekii* (louse-borne epidemic typhus) and *Rickettsia typhi* (murine typhus), which are transmitted by lice and infected flea feces, respectively [4,5,6]. TRG includes *Rickettsia felis* (flea-borne spotted fever), which are mainly transmitted by fleas [7], *Rickettsia australis* (Queensland tick typhus) transmitted by ticks [1], and *Rickettsia akari* (mite-borne rickettsialpox) transmitted by mites [6]. *Rickettsia bellii* and *Rickettsia canadensis* are considered non-pathogenic endosymbionts of ticks and belong to AG [8]. The SFG contains the most significant number of *Rickettsiae*, with more than 20 recognized species, including lethal ones such as *Rickettsia rickettsii* (Rocky Mountain spotted fever) and *Rickettsia conorii* (Mediterranean spotted fever) [9].

In recent years, the detection and identification of new species belonging to the genus *Rickettsia* has increased, largely due to the development and application of modern molecular techniques [10]. Currently, the genus *Rickettsia* has 31 validated species and more than 67 possible species that remain in candidate status [11]. Some of these new candidates for *Rickettsia* species have been involved as human pathogens, as was the case several years ago with *Candidatus* Rickettsia rioja, a species that has been involved in Dermacentor-borne necrosis erythema and lymphadenopathy (DEBONEL) or tick-borne lymphadenopathy (TIBOLA) cases from Spain [12]. Recently, *Candidatus* Rickettsia lanei was reported in two patients with Rocky Mountain spotted fever (RMSF-like) in California, USA [13]. This emergence of new pathogens, coupled with anthropogenic factors (such as loss of forest cover and biodiversity) [14] and climate change, causes the expansion and aggressiveness of the vectors. Rickettsiosis is considered a neglected disease that could put half of the world’s population at risk of contracting rickettsiosis from a species of SFG [15,16].

In 2012, a new candidate species of Rickettsia, *Candidatus* Rickettsia colombianensis, was described for the first time [17]. This Candidatus to Rickettsia was renamed to *Candidatus* Rickettsia colombiensis to comply with requirements of the International Code of Nomenclature of Prokaryotes [18]. *Candidatus* Rickettsia colombiensis has been detected in *Amblyomma dissimile* ticks collected from *Iguana iguana* and snakes in Córdoba, Colombia [17,19]; it has also been reported in ticks of the genera *Rhipicephalus* and *Dermacentor* sp. in different parts of Colombia [20,21,22,23,24]. Additionally, it has been detected in other countries in Central and South America [25,26,27,28,29].

In Colombia, the main vector of *Ca*. R. colombiensis is *A. dissimile*, with infection rates between 15% and 100% [17,19,23,30]. Given the high infection rate of this *Rickettsia*, there is a risk of transmission to humans and animals when bitten by this tick species. However, the pathogenicity of *Ca*. R. colombiensis in humans and animals is unknown. *Candidatus* Rickettsia colombiensis has the *ompA* gene, which is located in the SFG and is phylogenetically related to *Rickettsia tamurae* and *Rickettsia monacensis*, both of which are recognized as human pathogens [31,32].

Many *Rickettsiae* species discovered in ticks were initially described as endosymbionts because they were not associated with diseases in animals or humans [14]. However, some pathogens, such as *Rickettsia parkeri* and *Rickettsia slovaca*, were first described in ticks and were later identified as pathogens in humans and animals based on clinical records and microbiological approaches, indicating pathological findings [33,34]. All *Rickettsiae* species are thought to have the potential to be pathogenic in vertebrates. The limiting factor for the disease is the opportunity for transmission to vertebrate hosts [14,35].

In contrast, serological cross-reactivity between *Rickettsia* species of the same and different groups has been well documented [36]. *Ca*. R. colombiensis is a member of the SFG, so it would have cross-reactivity with other SFG members that circulate in the region and cause infections in humans and animals. This cross-reactivity could provide an opportunity for diagnosis in areas where laboratory supplies are scarce. In this regard, slides with *Ca.* R. colombiensis may aid in the diagnosis of rickettsiosis caused by SFG Rickettsiae.

Therefore, the aim of this study was to (i) evaluate the pathogenicity of *Ca*. R. colombiensis in a Syrian hamster (*Mesocricetus auratus*) model, (ii) assess IgG cross-reactivity of human sera from patients with SFG rickettsioses against *Ca.* R. colombiensis antigen, and (iii) assess IgG cross-reactivity of Syrian hamsters against other SFG rickettsial antigens.

## 2. Materials and Methods

### 2.1. Study Design and Tick Collection

In 2023, a prospective descriptive study was conducted at the Department of Córdoba. *Amblyomma dissimile* ticks were collected from iguanas (*Iguana iguana*) and snakes (*Boa constrictor*). Ticks were kept alive and transported to the Institute of Biological Research of the Tropics Laboratory, where they were taxonomically identified under a stereomicroscope (Leica EZ4HD, CH-9435, Heerbrugg, Switzerland), following the scheme described by Barros-battesti et al. [37] and stored at −90 °C.

### 2.2. Isolation and Culture of Candidatus Rickettsia Colombiensis

The shell vial culture was prepared as follows: a confluent monolayer of Vero cells in a 75 cm2 cell culture flask was used. Cells were removed with trypsin (Gibco, cat. No. 25200072, Waltham, MA, USA) and suspended in 10 mL of Dulbecco’s Modified Eagle’s medium (DMEM) (Gibco, cat. No. 11965092, Waltham, MA, USA) and transferred to a sterile conic tube. The suspension was centrifuged at 1500× *g* for 10 min and the pellet was re-suspended in 5 mL of new medium. Viable cells were counted in a Neubauer chamber (ThermoFisher Scientific, Waltham, MA, USA), and each shell vial was inoculated with approximately 200,000 cells and incubated at 37 °C without CO_2_ [38]. Ten *A. dissimile* specimens were cultured using the shell vial technique to isolate *Ca*. R. colombiensis. The ticks were disinfected with iodine alcohol for 10 min, washed with sterile water, and macerated in Brain Heart Infusion (BHI) broth (Thermo Scientific Oxoid, Waltham, MA, USA). The macerate (200 µL) was inoculated into shell vials containing Vero cells and supplemented with DMEM containing 5% bovine fetal serum (BFS) enriched with iron (Hyclone, Cat. No. SH30072-03, ThermoFisher Scientific, Waltham, MA, USA), 1% penicillin-streptomycin, and amphotericin B (Gibco, cat. No. 15240-062, ThermoFisher Scientific, Waltham, MA, USA) and incubated at 28 °C and 32 °C. Every three days, the shell vials were examined for the presence of *Rickettsia* using Giménez staining and PCR [38,39]. The positive shell vials were transferred to a 12 cm^2^ flask, and once more than 90% infection was achieved, the cells were transferred to a 75 cm^2^ flask. When >90% infection was obtained, the culture was used to inoculate Syrian hamsters and develop Indirect Immunofluorescence Assay (IFA) slides [38,40]. To determine the percentage of cell infection, 100 cells were observed in different random fields; it was determined how many had intracellular bacteria and the percentage of infection of the culture was calculated. One portion of the infected cells was cryopreserved in sucrose–phosphate–glutamate (SPG) buffer at −90 °C.

### 2.3. Animal Infection Model of Syrian Hamster (Mesocricetus auratus) with Candidatus Rickettsia Colombiensis

**Figure 1:**
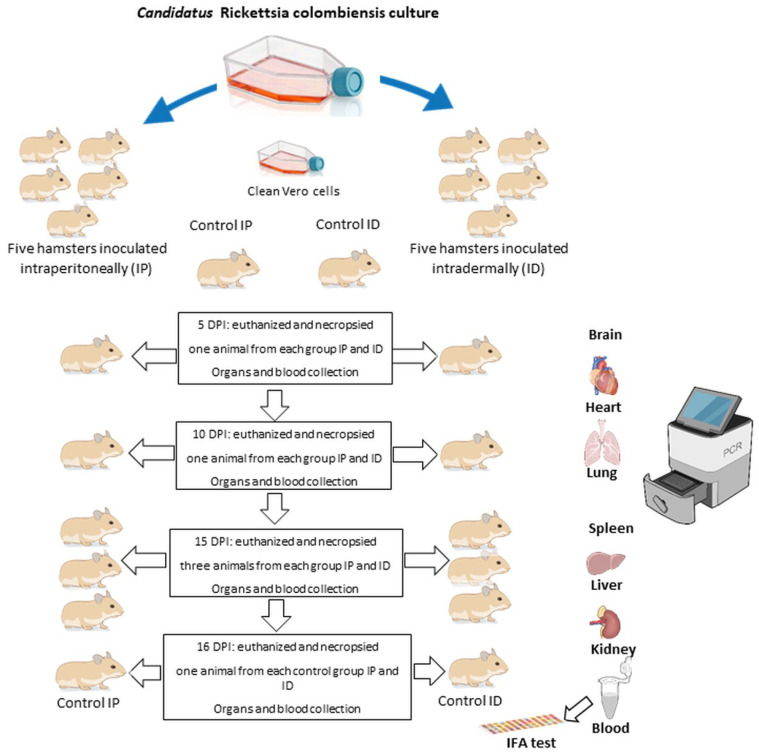
Flow diagram of inoculation and euthanasia of the 12 Syrian hamsters. DPIs: days post infection. IFA test: immunofluorescence assays. The image was created with NIH BIOART, accessed on 27 January 2026. (https://bioart.niaid.nih.gov/).

Twelve male Syrian hamsters aged 6 weeks and specific pathogen-free (SPF) hamsters were used in this study. Ten Syrian hamsters were included in the experimental group, and two were included in the control group. The infection method and evaluation of Syrian hamsters were modified according to previously described methods [41,42]. Five anesthetized Syrian hamsters were inoculated (day 0) intraperitoneally (IP group) with 500 µL of 1 × 10^6^ Vero cells infected with *Ca*. R. colombiensis isolated AdCor 5 third passage, and five Syrian hamsters were inoculated intradermally (ID group) with 1 mL of the same concentration of infected Vero cells with *Ca*. R. colombiensis in DMEM medium. Control hamsters (one for IP and one for ID) were inoculated with uninfected Vero cells. The number of Vero-infected cells was determined in a Neubauer chamber as follows: 450 µL of trypan blue (Gibco, cat. No. 1520061, ThermoFisher Scientific, Waltham, MA, USA) with 50 µL of infected cell suspension was mixed. After vortexing, 10 µL of the suspension was deposited on each side of the chamber and below a coverslip. All cells were counted using a microscope (Leica DMLS Fluorescence Microscope, Wetzlar, Germany) with a 10× objective. The total number of infected cells/mL was calculated, and the concentration was adjusted to 1 × 10^6^ infected Vero cells. A flowchart of the infection and euthanasia process is shown in Figure 1.

### 2.4. Clinical Monitoring and Histopathology

Syrian hamsters were evaluated daily for physiological parameters, including weight (w), temperature (°C), and mortality. Qualitative parameters were also analyzed, including the appearance of feces, urine, hair, skin, hydration level, behavioral changes, oral mucosa, and conjunctiva. The appearance of scrotal reactions, such as edema, congestion, and necrosis, which are typical of acute *Rickettsiae* infection in these animals, was also evaluated [43,44]. On day 5, one Syrian hamster from each group (IP and ID) was sacrificed, as well as on the 10 DPIs (days post infection). At 15 DPIs, three Syrian hamsters from each group were sacrificed, and at 16 DPIs, the controls hamsters were euthanized. Hamsters were randomly assigned to the sacrifice scheme.

Necropsies were performed to identify hemorrhages, necrotic lesions of the tissues, splenomegaly, testicular erythema, and lung petechiation [9,45]. Blood samples were drawn by cardiac puncture, and serum was assessed using IFA tests. The sera were analyzed using various *Rickettsia* antigens, including *Ca*. R. colombiensis, *R. rickettsii*, and *R. parkeri*. The organs were preserved, and qPCR, conventional PCR, and histological staining were performed. The euthanasia procedure is shown in Figure 1. The controls in each group were euthanized at 16 DPI. The following organs were collected at necropsy: brain, heart, lungs, spleen, liver, kidneys, and blood vessel fragments. Some of these tissues were frozen at −90 °C for qPCR, conventional PCR analysis, and culture. The remaining tissues were used for histopathological studies (10% buffered formalin). Organs were processed using standard histopathological protocols, and sections were prepared using hematoxylin and eosin (H and E).

### 2.5. Ethical Aspects

The Syrian hamsters were anesthetized with an intramuscular mixture of Ketamine (Ketalar^®^, Par Sterile Products LLC/Endo USA, Inc., Rochester, NY, USA) and Xylazine (10:1) (Rompun^®^, Elanco Animal Health, Greenfield, IN, USA), and a safe dose of the anesthetic combination of 0.1–0.3 mL was used for animals weighing 100–200 g. Euthanasia was performed using an overdose of sodium pentobarbital (Nembutal^®^, Akorn Pharmaceuticals, Gurnee, IL, USA), which was calculated according to the weight of the animal. The study was approved by the Institute of Biological Research of the Tropics Ethics Committee of the University of Córdoba Minutes 010-2022 on 12 October 2022. This study was approved by the University of Antioquia Ethics Committee for Animal Experimentation (minutes of session 151, 11 April 2023).

### 2.6. Preparation of Slides and Immunofluorescence Assays (IFA)

Cells with a level of infection ≥90% with *Ca*. R. colombiensis from the 75 cm^2^ flask were scraped off, transferred to a tube, and centrifuged (13,000× *g*/10 min). The pellet was resuspended in PBS containing 2% BFS and 0.1% sodium azide. Then, 10 ul of the infected cells was placed in each well of the slide and allowed to dry at room temperature in a biosafety cabinet, class II, Type A2 (NuAire, Plymouth, MN, USA). The antigen was fixed with acetone for 10 min and stored at −80°C until use [46]. Serodiagnosis by IFA test remains the gold standard for rickettsial infections using seroconversion and four-fold antibody titer increases in two paired sera separated by at least 2–6 weeks [47]. Briefly, each well of the slide contained *Ca*. R. colombiensis antigen, was covered with 10 µL of the hamster, and human sera was diluted 1:64 (10 µL of serum + 630 µL of PBS). The slides were then incubated at 37 °C for 30 min. The cells were then washed three times with PBS. Subsequently, 15 µL of conjugate was added; for Syrian hamster a 1:400 dilution of Goat Anti-Hamster IgG (H + L) and Mouse/Rat adsorbed-FITC was used (SouthernBiotech Cat. No 6061-02, Birmingham, AL, USA). For human sera, a 1:400 rabbit anti-human IgG (H + L)-FITC conjugate (MyBioSource Cat. No. MBS524382, San Diego, CA, USA) was incubated at 37 °C for 30 min. The slides were washed twice with PBS + Evans Blue (0.3 mL per 100 mL of PBS wash) [46]. The slides were observed under a fluorescence microscope (Leica DMLS Fluorescence Microscope, Wetzlar, Germany) at 400×. Serum was considered to contain antibodies against *Rickettsia* if it displayed a bright green color on extracellular or intracellular coccobacilli fixed to the slide at a 1:64 dilution. In each slide, a serum previously shown to be non-reactive (negative control) and a known reactive serum (positive control) were used.

### 2.7. Cross-Reactivity of Syrian Hamsters and Human Serum

Cross-reaction refers to the ability of antibodies elicited against antigens of one *Rickettsia* species to bind to and react with antigens from other *Rickettsia* species, because of the presence of shared or structurally similar antigenic epitopes. This immunological phenomenon is common among closely related *Rickettsia* species, particularly within the spotted fever group [48,49]. Serological cross-reactions between *Rickettsiae* species and other bacterial genera can occur because of the production of antibodies that recognize proteins with the same antigenicity [50]. For the purposes of this study, a serum was considered cross-reactive if it showed IgG reactivity at a titer ≥1:64 to more than one SFG rickettsial antigen in the IFA test.

IFA test can help in the identification of rickettsial species; if it demonstrates a fourfold higher dilution compared to other rickettsial antigens, this may be suggestive of a causative microorganism. However, cross-reactions or the maintenance of high antibody titers for a long time can interfere with this technique [51]. To establish the degree of cross-reactivity of the antibodies in Syrian hamsters and human sera infected previously with *Rickettsia* sp. of the SFG, slides prepared with *R. rickettsii*, *R. parkeri*, and *Ca*. R. colombiensis was used in this study. Slides with *R. rickettsii* and *R. parkeri* were donated by Professor Marcelo Labruna of the Universidad São Paulo, Brazil.

### 2.8. Human Sera for Cross-Reactivity Study of Rickettsia sp. in the Spotted Fever Group

A total of 150 serum samples were obtained from the serum bank at the Institute of Biological Research of the Tropics. These samples were obtained from seroprevalence and outbreak studies conducted between 2007 and 2015 in different areas of the Cordoba department in Colombia. Informed consent and ethical approval were obtained at the beginning of these studies. All participants were diagnosed with *Rickettsiae* infection of the SFG [52,53]. Four percent of these subjects (6/150) had confirmed infection with *R. rickettsii* (by IFA, PCR, and sequencing). The remaining 96% (144/150) of the participants underwent seroprevalence studies with positive IFA results. Slides with *R. rickettsii* antigens were used to detect antibodies against SFG rickettsia in human serum.

### 2.9. Molecular Detection Rickettsia by qPCR and PCR Conventional

DNA was extracted from Syrian hamster sera, tissues, and cultures using the PureLinK Genomic DNA Purification Kit (Ref: 1820-00, Invitrogen, ThermoFisher Scientific, Waltham, MA, USA). qPCR was performed with a TaqMan probe specific for the *Rickettsiae* genus using primers CS-5 and CS-6, targeting a 147 bp fragment of the citrate synthase (*gltA*) gene [38]. The assay was performed using 10 µL of GoTaq Probe qPCR Master Mix (Promega Cat. No A6101, Madison, WI, USA), 1 µL of each primer at 10 µM, and 0.5 µL of *Rickettsia* probe at 20 µM (FAM-BHQ1) (Macrogen Inc. Seoul, South Korea). For each qPCR, 5 μL of human DNA was used as a negative control, molecular grade water as a non-template control, and *Rickettsia amblyommatis* DNA was used as a positive control. Human RNAseP was used as internal amplification control, probe label (Quasar 670-BHQ2) (Macrogen Inc. Seoul, South Korea) [54]. PCRs were performed in a CFX96 thermal cycler (Bio-Rad Laboratories, Hercules, CA, USA), and the cycling conditions were as follows: 1 cycle at 95 °C for 2 min, followed by 40 cycles of 15 s at 95 °C and 1 min at 60 °C. Samples were considered positive when the Ct ≤ 37 cycle. The limit of detection reported in the validation of this qPCR was determined to be 1 DNA copy of *R. rickettsii* and 100 DNA copies of *Rickettsia bellii*.

For detection and molecular characterization to confirm the identity and the phylogenetic position in the SFG of *Ca*. R. colombiensis isolates, a fragment of the *ompA* gene was amplified using primers Rr 190.70 and 190-701 [55]. The assay was performed using 0.5 µL of Taq DNA Polymerase, recombinant (Invitrogen Cat. 11615-036, Carlsbad, CA, USA), 5 µL of 10× PCR Buffer, minus Mg, 1.5 µL of 50 mM MgCl_2_, 2.5 µL of each primer at 10 µM, and 1.0 µL of 10 mM dNTP mix. For each PCR, 5 μL of human DNA was used as a negative control, molecular grade water as a non-template control, and *Rickettsia amblyommatis* DNA was used as a positive control. PCRs were performed in a T100 PCR thermal cycler (Bio-Rad Laboratories, Hercules, CA, USA), and the cycling conditions were as follows: 1 cycle at 95 °C for 3 min, followed by 40 cycles of 30 s at 95 °C, 30 s at 55 °C, 60 s at 72 °C, and 7 min at 72 °C. PCR products were separated by electrophoresis on a 1.5% agarose gel, stained with SYBR safe, and examined using an ultraviolet (UV) transilluminator Molecular Imager Gel Doc™ XR+ gel documentation system (BIO-RAD Laboratories, Hercules, CA, USA).

To confirm infection by *Ca*. R. colombiensis, the amplicons were purified with NucleoSpin Gel and PCR Clean Up (Ref: 720609.50, MACHEREY-NAGEL GmbH & Co. KG, Düren, Germany) and both chains were sequenced using the dideoxy method in a SeqStudio, (Applied Biosystems by Thermo Fisher Scientific, Carlsbad, CA, USA) in the Research Centre of Rickettsiosis and Arthropod-Borne Diseases (CRETAVs), Infectious Diseases Department, San Pedro University Hospital—Center for Biomedical Research (La Rioja, Spain).

### 2.10. Phylogenetic Analysis

To confirm the identity and the phylogenetic position of *Ca*. R. colombiensis in the SFG, a partial *ompA* gene sequence was used. This sequence was aligned with other 22 *Rickettsiae* sequences available in GenBank (Table S1) using MEGA X [56]. Phylogenetic trees were constructed using the maximum likelihood (ML) method, implemented in IQ-TREE Web Server version 1.6.12 [57], which automatically selected the best-fitting evolutionary model through ModelFinder according to the Bayesian Information Criterion (BIC). K3Pu + F + G4, corresponding to the Kimura three-parameter model with unequal substitution rates, empirical base frequencies, and a gamma distribution with four rate categories, was the best nucleotide substitution model for the phylogenetic trees. The reliability of the analysis was determined using bootstrap analysis with 1000 ultrafast bootstrap (UFBoot) replicates. The phylogenetic tree was visualized and edited using FigTree version: 1.4.4 (http://tree.bio.ed.ac.uk/software/figtree/, accessed on 27 January 2026).

### 2.11. Data Analysis

The results obtained for physiological parameters (weight and temperature) and antibody titers were recorded in Microsoft Excel LTSC MSO (version: 16.0.14334.20440), (Microsoft Corp., Redmond, WA, USA) for tabulation and analysis. Due to small sample size and exploratory nature of the study, only descriptive statistics were performed and no formal hypothesis testing was carried out.

## 3. Results

### 3.1. Isolation of Candidatus Rickettsia Colombiensis

**Figure 2:**
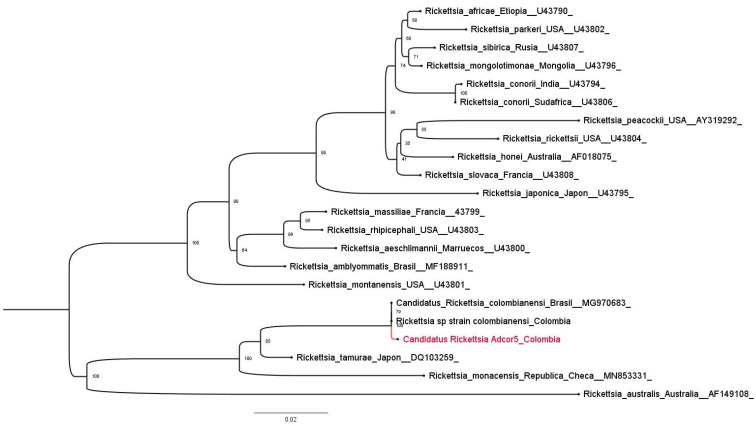
Phylogenetic position of *Ca*. R. colombiensis AdCor5 (in red) among the validated SFG rickettsial species. The analysis included 22 partial *ompA* nucleotide sequences from different SFG *Rickettsia* species. The country of origin and GenBank accession number for each sequence are shown in the phylogenetic tree. All positions containing gaps and missing data were removed. The final dataset contained 564 positions.

Two isolates of *Ca*. R. colombiensis (AdCor5 and AdCor6) were also established. The *gltA* gene was detected in both isolates using qPCR (primers CS5 and CS-6) with cycle threshold (ct) of 23 and 25 for AdCor5 and AdCor6, respectively. The *ompA* gene (632 bp fragment) was detected using conventional PCR. Assays and phylogenetic analyses were performed using *Ca*. R. colombiensi isolate AdCor5. Sequencing of the amplified *ompA* products showed 100% nucleotide identity with the *Rickettsia* sp. strain Colombianensi (JF905458). Phylogenetic analysis of partial *ompA* sequence revealed that *Ca*. R. colombiensis isolate AdCor5 was grouped with other reported *Ca*. R. colombiensis sequences detected in Colombia and Brazil at a bootstrap level of 100%. Furthermore, phylogenetic analysis showed that isolate AdCor5 was grouped in the same clade as *R. tamurae* and *R. monacensis*, with high bootstrap levels of 99%, as showed in Figure 2. The nucleotide sequence of the *ompA* gene fragment of AdCor5 was deposited in GenBank under accession number PQ658084 (https://www.ncbi.nlm.nih.gov/nuccore/PQ658084.1/) (accessed on 27 January 2026).

### 3.2. Evaluation of the Pathogenesis of Ca. R. Colombiensis in an Experimental Syrian Hamster (M. auratus) Model

**Figure 3:**
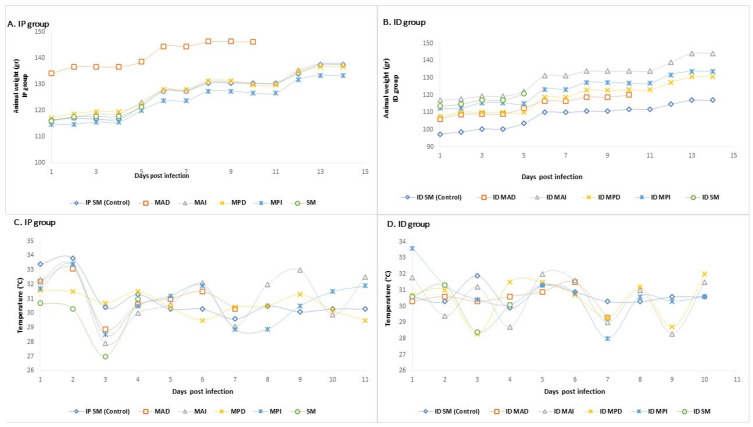
Weight measurements for both groups of *Ca*. R. colombiensis-infected Syrian hamsters (**A**,**B**). Temperature measurements in both groups (**C**,**D**). IP: intraperitoneal; ID: intradermal.

All Syrian hamsters (12) remained healthy during the trial until they were euthanized. They did not show changes in quantitative physiological parameters, such as weight loss, high temperature, and mortality. Syrian hamsters in the IP group showed an average weight gain of 16 g (5.3–21 g). The ID group showed an average weight gain of 18.7 g (7–19.7 g). Figure 3A,B show the parameters of body weight and Figure 3C,D the daily recorded temperature of the Syrian hamsters. No abnormal changes were observed in the feces, urine, hair, skin, hydration level, behavior, appearance of the skin, oral mucosa, or conjunctiva, and no reactions such as edema, congestion, or necrosis were observed in the scrotum. The Syrian hamsters in the control group did not exhibit any signs of disease or pathological changes associated with rickettsial infection. No hemorrhage, vasculitis, or tissue necrosis was observed during necropsy. Histopathological analysis of the liver, lung, brain, spleen, aorta, and kidney tissues stained with hematoxylin and eosin revealed no lesions associated with rickettsial infection. However, some organs, such as the liver and kidney, presented lesions, such as moderate multifocal lymphocytosis in the interstitial tubule, lymphocytosis, focal membranoproliferative glomerulopathy, and mild multifocal periportal plasmacytosis in the liver (Figure S1). In the case of control group animal, moderate to severe chronic diffuse granulomatous interstitial pneumonia and focal membranoproliferative glomerulonephritis were observed.

### 3.3. Detection of Rickettsiae in Syrian Hamster Tissues

DNA was extracted from each organ (blood, serum, liver, lung, brain, spleen, and kidney) of the 12 Syrian hamsters. A total of 84 DNA extractions were obtained, all of which were negative for *Rickettsia* sp. by qPCR and conventional PCR (*ompA*).

### 3.4. Indirect Immunofluorescence (IFA) on Syrian Hamster Serum

**Figure 4:**
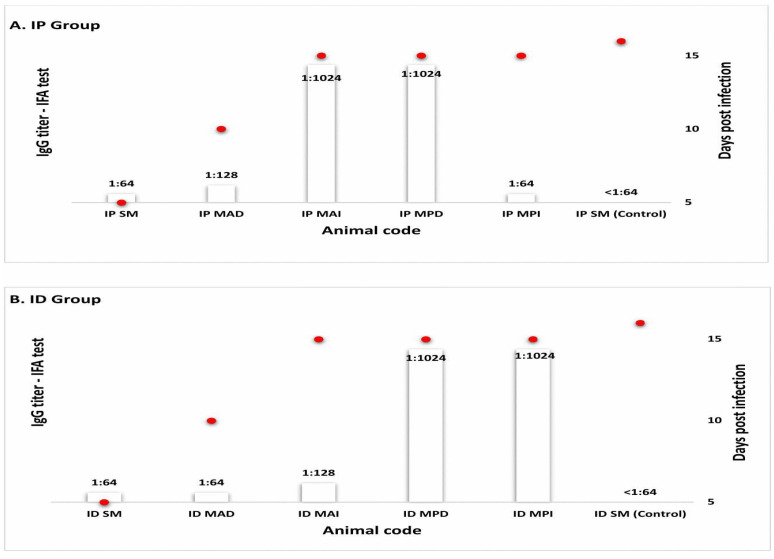
(**A**) Anti-*Ca*. R. colombiensis IgG endpoint titers of Syrian hamster in the IP (intraperitoneal) group inoculated with *Ca*. R. colombiensis.; (**B**) Anti-*Ca*. R. colombiensis IgG endpoint titers in the ID (intradermal) group. The red dots indicate the day the animals were sacrificed.

Twelve serum samples from Syrian hamster were analyzed in this study. All sera from IP and ID inoculated Syrian hamsters showed anti-*Ca*. R. colombiensis IgG antibodies. They were detected starting at 5 DPIs with titers of 1:64. The maximum titer was 1:1024 at 15 DPIs. The control Syrian hamsters did not exhibit any anti-*Ca*. R. colombiensis antibodies. The antibody titers in each inoculation group are shown in Figure 4.

### 3.5. Cross-Reactivity in Syrian Hamster and Human Sera

**Figure 5:**
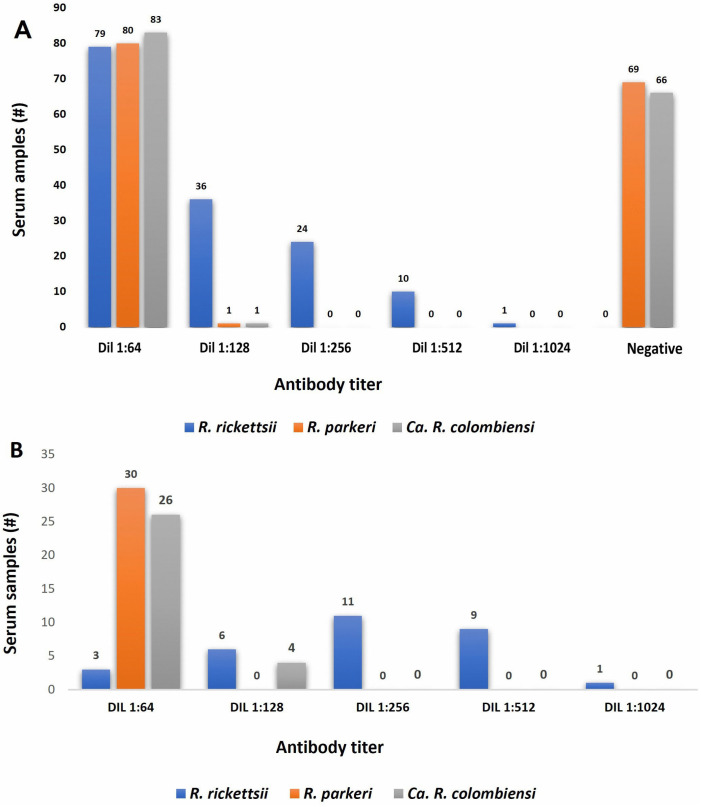
(**A**) Numbers and antibody titers of human sera tested against *R. rickettsii*, *R. parkeri*, and *Ca*. R. colombiensis antigens in 150 subjects. (**B**) Number and antibody titers of human sera from 30 subjects.

None of the sera from the 12 Syrian hamsters (10 from the IP- and ID-infected Syrian hamsters and two controls from each group) presented antibodies against *R. rickettsii* or *R. parkeri* antigens. All 10 serum samples from the infected Syrian hamsters presented with antibodies against *Ca*. R. colombiensis antigen, with titers between 1:64 and 1:1024. Regarding human sera, initially 56% (84/150) had IgG antibodies against *Ca*. R. colombiensis antigen, demonstrating cross-reactivity. The same sera were tested for *R. parkeri*, showing 54% (81/150) of cross-reactivity. Figure 5A shows the number and antibody titers of human sera tested against *R. rickettsii*, *R. parkeri*, and *Ca*. R. colombiensis antigens. Subsequently, of the 150 sera tested, 30 were selected with titers equal to or greater than 1:64, distributed as follows: three sera with titers of 1:64, six sera with titers of 1:128, eleven sera with titers of 1:256, nine sera with titers of 1:512, and one serum with a titer of 1:1024. This selection was made because 52% of the sera (79/150) had an antibody titer of 1:64. A low titer of 1:64 did not indicate acute or recent infection; therefore, these patients were excluded. All 30 samples were 100% seroreactive for *Ca*. R. colombiensis antigen. However, the titers of *R. rickettsii* and *Ca*. R. colombiensis were differentially expressed. Figure 5 shows the titers obtained from the analysis of these samples (Figure 5B).

## 4. Discussion

In the present study, anti-*Ca*. R. colombiensis IgG antibodies were detected in Syrian hamsters; however, they did not show signs of disease similar to the one caused by pathogenic *Rickettsia*. We reported that some Syrian hamsters presented with lesions in the histological sections of the kidney and liver. Since no vasculitis-related rickettsiosis was identified in tissues, these lesions could indicate lesions of a genetic background that are frequent in rodents used for research purposes and are possibly not associated with the pathological process of the infection [58] (Figure S1). According to NTP Nonneoplastic Lesion Atlas, inflammatory lesions associated with aging are commonly found in animals older than 28 or 90 days [58], which agrees with our findings, since the age of the hamsters used in our study was 42 days (6 weeks). The skill of the pathologist was used to decide whether or not the lesions were associated with age-chronic inflammation or with Ca. R. colombiensis infection. These results, combined with the negative PCR results, confirm the pathologist’s findings.

Animal models can imitate the complex pathogenesis of infections [59]. In this sense, different animal species have been used as models for studying the pathogenesis of *Rickettsiae*. Guinea pigs (*Cavia porcellus*) have been the most widely used [43,60]. C3H/HeN, C57BL/6, Balb/c mice, and Syrian hamsters (*M. auratus*) have been used as models for pathogenesis studies [61,62,63]. The Syrian hamster model used in the present study has been reported as one of the best models for pathogenesis studies because of its physiological similarity to the human immune system compared with that of other animal species [59].

Human pathogenic *Rickettsia*, such as *R. prowazekii* and *R. rickettsii*, causes lethal infections or diseases in guinea pigs [60]. The first studies on pathogenicity proposed the hypothesis that the infection and lethality rates of *Rickettsia* spp. observed in guinea pigs could be an extrapolated model to assume the possible infection and lethality in humans. However, McDade [64] reported no correlation between animal and human fatalities and suggested that this extrapolation should be performed cautiously.

To our knowledge, this is the first study to evaluate the pathogenicity of *Ca*. R. colombiensis in a Syrian hamster model. Other *Rickettsia* species have also been reported to be non-pathogenic in vectors and have been subsequently assessed in animal models. In this regard, *Rickettsia amblyommatis* (formerly *Candidatus* Rickettsia amblyommii) was initially classified as non-pathogenic because no signs of the disease were observed in guinea pigs [65,66]. However, in both studies, an IgG-type immune response was observed, indicating subclinical infection. These results agree with those reported in our study, where no clinical or histopathological signs of the disease were observed, but an evident antibody response was detected. In previous studies on guinea pigs, *R. amblyommatis* was considered non-pathogenic [65,66]. However, recent studies [9,45] have reported clinical findings such as bilateral enlargement of the testicles and histopathological findings such as inflammatory processes around the testis and necrotic lesions in the liver. In addition, an IgG-type immune response was observed, indicating an infection similar to that caused by pathogenic *Rickettsia* of the SFG. These contradictory results could be explained by the fact that the following three different strains of *R. amblyommatis* were used, isolated from other sites in the United States: strain WB-8-2T [66], North Texas isolates [65], Lake Alexander strain [9], and strain 9-CC-3-1 isolated from Costa Rica [45].

Another *Rickettsiae* species of unknown or controversial pathogenicity that has been evaluated in animal models to determine its pathogenicity is *Rickettsia bellii*. This species is considered non-pathogenic. The concept of non-pathogenicity is based on the fact that it does not cause disease in guinea pigs or *Microtus pennsylvanicus* [67]. These results are similar to those of another study in which no clinical signs were observed in guinea pigs or opossums (*Didelphis aurita*) infected with *R. bellii* [68]. However, high titers of anti-*R. bellii* antibodies were detected. These data are consistent with those reported in the present study for *Ca*. R. colombiensis, which did not produce clinical signs but did produce an immunological response, indicating subclinical infection.

*Rickettsia monacensis* was initially detected only in *Ixodes ricinus* in several European countries. In an animal model of six Syrian hamsters injected intraperitoneally with *R. monacensis*, all the animals remained healthy during the study. However, five seroconverted with a high titer of 1:16,384 demonstrated subclinical infection [63]. These results are similar to those of the present study, in which we used the same animal model, but none of the animals presented with signs. Nevertheless, the animals developed an immune response against *Ca*. R. colombiensis antigen. It is important to note that *R. monacensis* is currently recognized as a human pathogen [69].

The subclinical infection caused by *Ca*. R. colombiensis, as well as the absence of DNA in the tissues of sacrificed animals after 5 DPI, could suggest that this candidatus species did not behave as a highly virulent pathogen in Syrian hamsters. It is possible that the findings compatible with a lower pathogenic potential in this model of the isolated AdCor5 are affected by the absence of some virulence genes not studied in this research. The loss of virulence genes can lead to decreased pathogenicity as it was demonstrated by Kleba et al., who disrupted the Sca2 gene and inhibited the actin-based motility required for intracellular movement of *R. rickettsii* [70].

The possible low pathogenicity of *Ca*. R. colombiensis may have epidemiological implications because of its protective cross-reactivity exclusion against pathogenic Rickettsia species. This was proposed by Rivas et al. [45], who reported protective cross-immunity between *R. amblyommatis* and *R. rickettsii*, which may have implications for the epidemiology and severity of infections caused by SFG species in areas where several species circulate. Another important aspect that could have implications for the ecology of rickettsiosis in the region is the high infection rates in *A. dissimile* ticks of *Ca*. R. colombiensis has, which could interfere with the colonization of other *Rickettsia* species in ticks, as reported by Krawczak et al. [71], who proposed that the high rate of *Candidatus* R. andeanae may promote the exclusion of *R. parkeri* in *A. tigrinum* ticks.

The results of the present study demonstrated that in a selected subset of 30 sera with moderate to high antibodies titers induced by pathogenic rickettsiae of the SFG (*R. rickettsii* and *R. parkeri*), all samples (100%) reacted with *Ca*. R. colombiensis antigen, consistent with strong cross-reactivity among SFG antigens. The cross-reactivity observed in the present study was similar to that found in human sera using IFA conducted in an endemic area of Colombia [72]. In that study, a cumulative incidence of 6.23% (17/273) was reported after one year of follow-up. IFA was performed with antigens from *R. parkeri*, *R. rickettsii*, *R. amblyommatis*, *R. belli*, *R. rhipicephali*, and *R. felis* to establish the species of *Rickettsiae* involved. However, it was impossible to establish the probable species responsible for human infection because all 17 subjects had cross-reactivity against the six antigens used. Only three participants showed titers higher than 1:1024 (two participants with a titer of 1:2048 for *R. rickettsii* and *R. amblyommatis* and one participant with a titer of 1:1024 for *R. rickettsii* and *R. amblyommatis)* (Table 1).

Other studies that assess cross-reactivity by IFA with results similar to those our study were reported by Delisle et al. [73] and Vaughn et al. [74] in USA (Table 1). In these studies, a 100% cross-reactivity was found between the different antigens of *Rickettsia* sp. The main conclusion of these studies was that *R. rickettsii* is not the only cause of rickettsiosis in the study areas. The application of techniques such as cross-adsorption and Western blotting of rickettsial antigens revealed the exposure to species such as *R. montanensis*, *R. parkeri*, and *R. amblyommatis* as a common agent of spotted fever. Although it was not the purpose of this study to establish the etiological agent that caused the infection in the subjects, it is important to consider that IFA has limitations in identifying the infecting species, even when the samples are paired. Western blotting and cross-adsorption assays may overcome this issue; however, these methods are expensive, require technical expertise, and are limited to reference laboratories [51].

Travelers returning from different parts of the world are exposed to diseases caused by *Rickettsiae* endemic to the country. In countries such as the United States, the most frequent rickettsiosis among travelers (90% of cases) is the African tick-bite fever, caused by *Rickettsia africae*. However, in the USA, there are no specific tests for *R. africae*, and most available tests use antigens from *R. conorii* or *R. rickettsii* [75]. Although the antigens are different, cross-reactivity between the *Rickettsiae* of the SFG is evident, making them helpful in the diagnosis of *R. rickettsii* infection [36].

Regarding serological cross-reactivity in the Syrian hamsters, we did not find antibodies that cross-reacted with other species of SFG *Rickettsiae* at 16 DPI. These data contrast with those of Simser et al. [63], who reported the cross-reactivity of *R. monacensis*, *Rickettsia peacockii*, *R. rickettsii*, and *Rickettsia helvetica* in Syrian hamsters. In that study, sera were collected 50 days post-infection, and the antibodies reached a titer of 1:16.384. In our study, anti-*Ca*. R. colombiensis IgG titers ranged from 1:64 to 1:1024, and there was no cross-reactivity between *R. rickettsii* and *R. parkeri* species. A possible biological explanation for these results is that the hamster’s immune system recognizes different *Rickettsia* immunodominant epitopes or that the specific *Ca*. R. colombiensis strain elicits a highly specific response in this host. Another possible explanation is that the infection time in our study was 16 DPIs, which did not allow for the expression of heterologous antibodies that react with common antigens among the *Rickettsiae* species. Perhaps by prolonging the post-infection period, the appearance of these antibodies would have been possible.

A study on the mechanisms of cell-mediated immunity demonstrated that guinea pigs were protected against *R. rickettsii* after infection by *R. rhipicephali* [76]. The study reported that 100% of the animals in the three groups infected with *R. rhipicephali* at different doses (low: 7.5 × 10^2^ PFU, medium: 7.5 × 10^4^ PFU, and high: 7.5 × 10^6^ PFU) had antibodies that cross-reacted with *R. rickettsii* from 10 DPIs. These results contrast with those reported in our study, in which no cross-reactivity was observed with other SFG species (*R. rickettsii* and *R. parkeri*). We believe that several variables, such as the animal model used (Syrian hamster) and the time of exposure to infection (16 DPIs), partly explain the difference in the results.

In our study, Syrian hamsters showed anti-*Ca*. R. colombiensis IgG antibodies on day 5 of DPIs. These results are similar to those obtained by Blanton et al. [65], who reported IgG seropositive guinea pigs on day 7 of DPIs, with titers ranging from 1:64 to 2048 in animals inoculated with *R. amblyommatis*. Our results also agree with those reported by Rivas et al. [45], who reported anti-*R. amblyommatis* IgG antibodies in guinea pigs on day 7 of DPIs. However, our results differ from those reported by Snellgrove et al. [9], where all animals remained seronegative until day 13 of DPIs. Most authors report that IFA assays are insensitive during the first week of rickettsial infection [47,51,75]; however, this criterion has been adopted from human diagnostics, as reported by Stokes et al. [42]. This implies that better standardization criteria must be established for the animals. In contrast, the early detection of IgG in Syrian hamsters could represent a very robust immune response that should be established in future studies.

The reported experience with *Rickettsia* species that were initially described in the vector and did not cause disease in humans but were later established to be pathogenic demonstrates that further studies are needed, such as experimental transmission trials by ticks to mammalian hosts and antibody titer assessments in subjects, which will be essential to fully determine the pathogenic potential of *Ca*. R. colombiensis. One of possible weaknesses of our study was the lack of quantification of the infectious dose in PFU or genome copies, which means that true infective dose remains unknown and could imply heterogeneity in the immune response and pathogenic severity in Syrian hamsters. Another possible weakness is that we did not evaluate the sera with serum cross-adsorption or Western blotting to establish the species of *Rickettsia* involved in the infection of the sera used. Another possible weakness of this study is the short duration (16 days) of infection with *Ca*. R. colombiensis in hamsters. Increasing the infection time could lead to an increase in the number of antibodies, including heterologous antibodies.

In conclusion, the partial *ompA* sequence of isolate AdCor5 showed 100% nucleotide identity with others published *Ca*. R. colombiensis sequences, supporting its classification within *Ca*. R. colombiensis specie. Under the specific conditions of this study (10 infected Syrian hamsters, inoculation doses, inoculation routes, and a 16-day observation period), *Ca*. R. colombiensis did not induce overt clinical disease or typical histopathological lesions and no rickettsial DNA was detected in hamsters, although all inoculated animals developed IgG antibodies, suggesting the possibility of infection in other mammalian hosts. However, further studies are needed to draw definitive conclusions. As previously known, rickettsial cross-reactivity makes it difficult to diagnose a specific species that causes rickettsiosis, although it could be useful for confirming rickettsial syndrome.

## Figures and Tables

**Table 1:** Serological reaction studies between SFG species in different sites in South and North America.

Presumptive Infection *Rickettsia*/(n=) /Country	*Rickettsia* Cross-Reaction	Samples with Cross-Reaction (%)	Confirmed by Cross-Adsorption and Western Blot	References
*R. rickettsii*/(n = 76) /USA	*R. parkeri-R. montanensis-R. amblyommatis-R. rickettsii*	66	35 *R. amblyommii*, 1 *R. montanensis*, 1 *R. parkeri* 39 indeterminate 0 *R. rickettsii*	[65]
	*R. montanensis*, *R. parkeri*, *R. amblyommatis*	9		
	*R. parkeri*, *R. amblyommatis*	7		
	*R. rickettsii*, *R. montanensis*, *R. parkeri*	3		
	*R. rickettsii*, *R. montanensis*	2		
	*R. montanensis*, *R. amblyommatis*	2		
	*R. rickettsii*, *R. montanensis*, *R. amblyommatis*	1		
	*R. rickettsii*, *R. parkeri*, *R. amblyommatis*	1		
	*R. amblyommatis*	5		
SFG rickettsiosis/(n = 17) /Colombia	*R. rickettsii*, *R. amblyommatis*, *R. felis*, *R. parkeri*, *R. rhipicephali*, *R. belli*	14	ND	[66]
	*R. rickettsii*, *R. amblyommatis*, *R. felis*, *R. belli*	3		
*R. rickettsii*/(n = 21) /United States	*R. rickettsii R. parkeri R. amblyommatis*	7	2 *R. parkeri* 2 *R. rickettsii* 1 *R. amblyommii* 2 indeterminate	[67]
	*R. parkeri y R. amblyommatis*	3		
	*R. rickettsii*	1		
	*R. parkeri*	4		
	*R. amblyommatis*	6		
*R. rickettsii*/(n = 30)/Colombia	*R. rickettsii*, *R. parkeri*, *Ca.* R. colombiensis	30	ND	Present study

## Data Availability

The data supporting the results and conclusions of this article are contained within this article.

## Supplementary Materials

The following supporting information can be downloaded at https://www.mdpi.com/article/10.3390/pathogens15020146/s1, Table S1: *Rickettsia* species and the GenBank accession number used to construct the phylogenetic tree of partial *ompA* gene. Figure S1: Histopathological analysis of different hamster tissues. A and B correspondent to lesions of a genetic background normally found in the kidney and liver. (A) lesions in the kidney: focal membranoproliferative glomerulopathy and moderate multifocal lymphocytosis in the interstitial tubule. (B) mild multifocal periportal plasmacytosis in the liver. C to H correspondent to histopathological analysis animal control tissues.

## Abbreviations

The following abbreviations are used in this manuscript:

Ca	Candidatus
Ct	Cycle threshold
IP	Intraperitoneally
ID	Intradermally
DNA	Deoxyribonucleic acid
qPCR	Quantitative or real-time PCR
SFG	Spotted fever group
TG	Typhus group
TRG	Transitional group
AG	Ancestral group
BHI	Brain Heart Infusion
DMEM	Dulbecco’s Modified Eagle’s medium
BFS	Bovine fetal serum
IFA	Indirect Immunofluorescence Assay
SPG	Sucrose–phosphate–glutamate
SPF	Specific pathogen-free
AdCor 5	*Amblyomma dissimile* (isolated number 5)
w	Weight
°C	Temperature
H&E	Hematoxylin and eosin
PBS	Phosphate-buffered saline
H + L	Heavy- and light-chain IgG
FITC	Fluorescein isothiocyanate
*gltA*	Citrato sintasa (glutamate metabolism)
*OmpA*	Outer membrane protein A
