# Supplementary material for: Assessment of the Pathogenicity of Candidatus Rickettsia Colombiensis in a Syrian Hamster Model and Serological Cross-Reactivity Between Spotted Fever Rickettsia Species
Source: Pathogens. 2026 Jan 29;15(2):146. doi: 10.3390/pathogens15020146 (PMC12943140; doi:10.3390/pathogens15020146)
Supplement: Supplementary file 1 [file pathogens-15-00146-s001.zip › Figure S1.pdf]

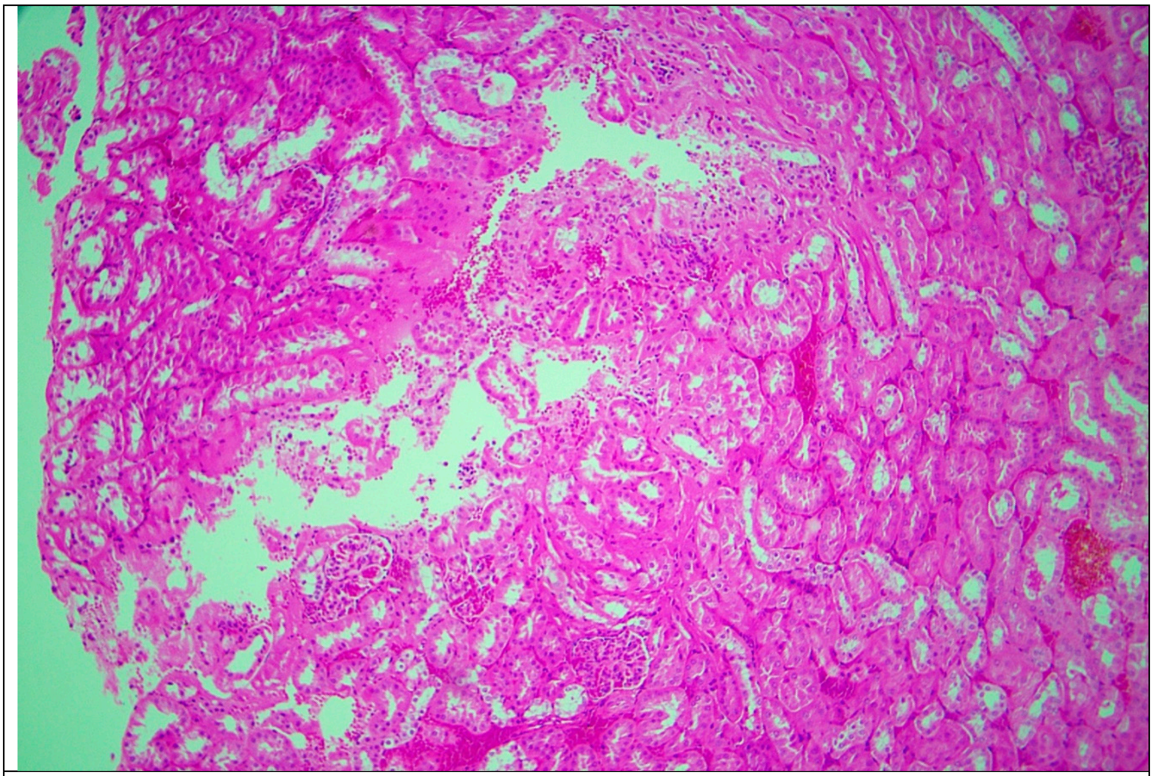

A

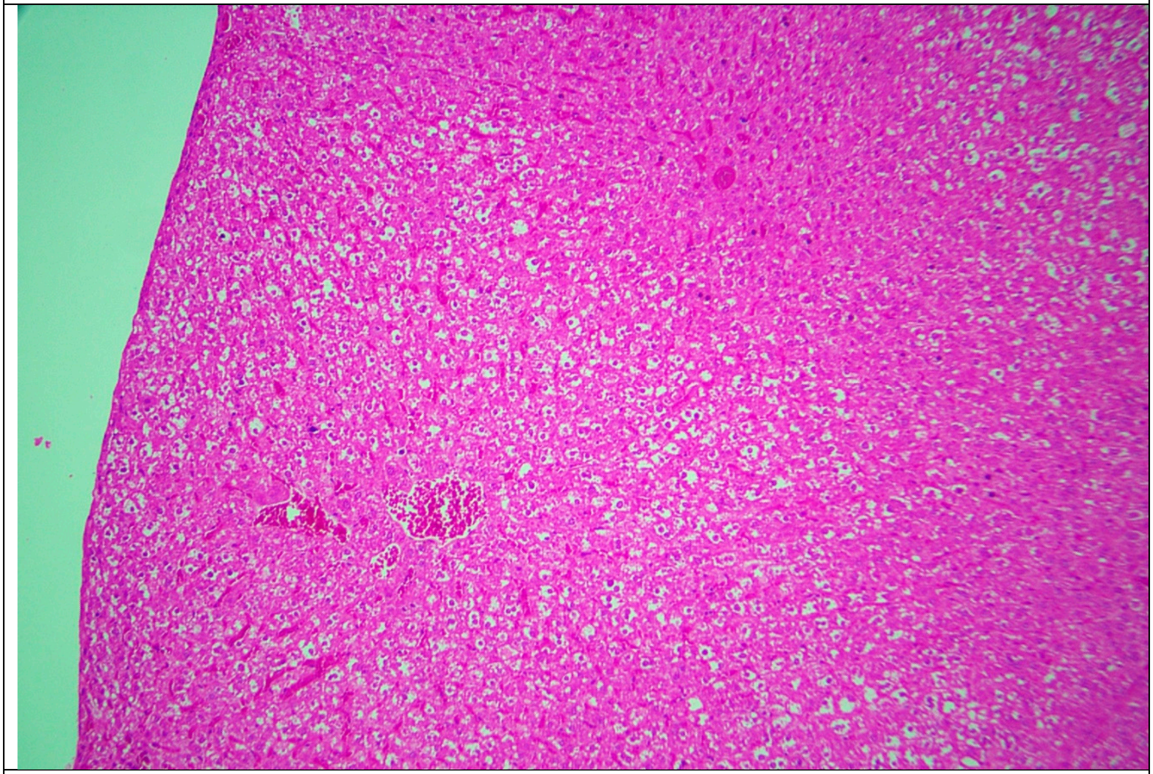

B

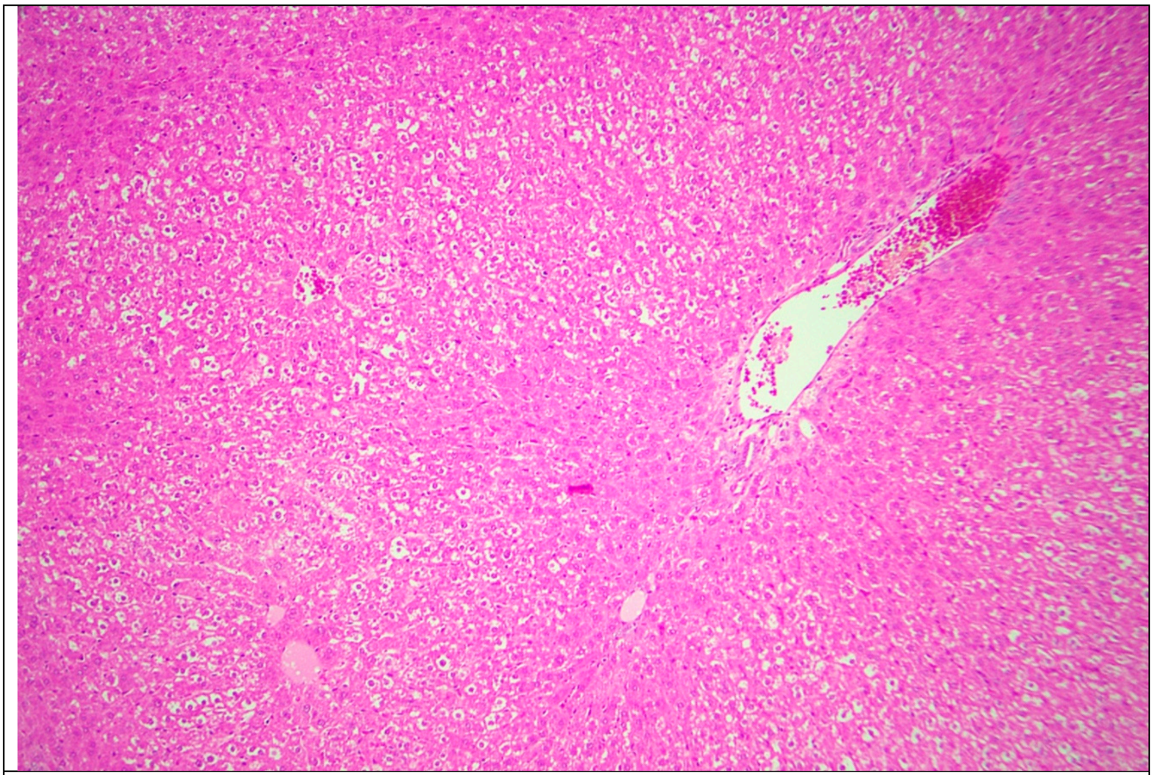

C

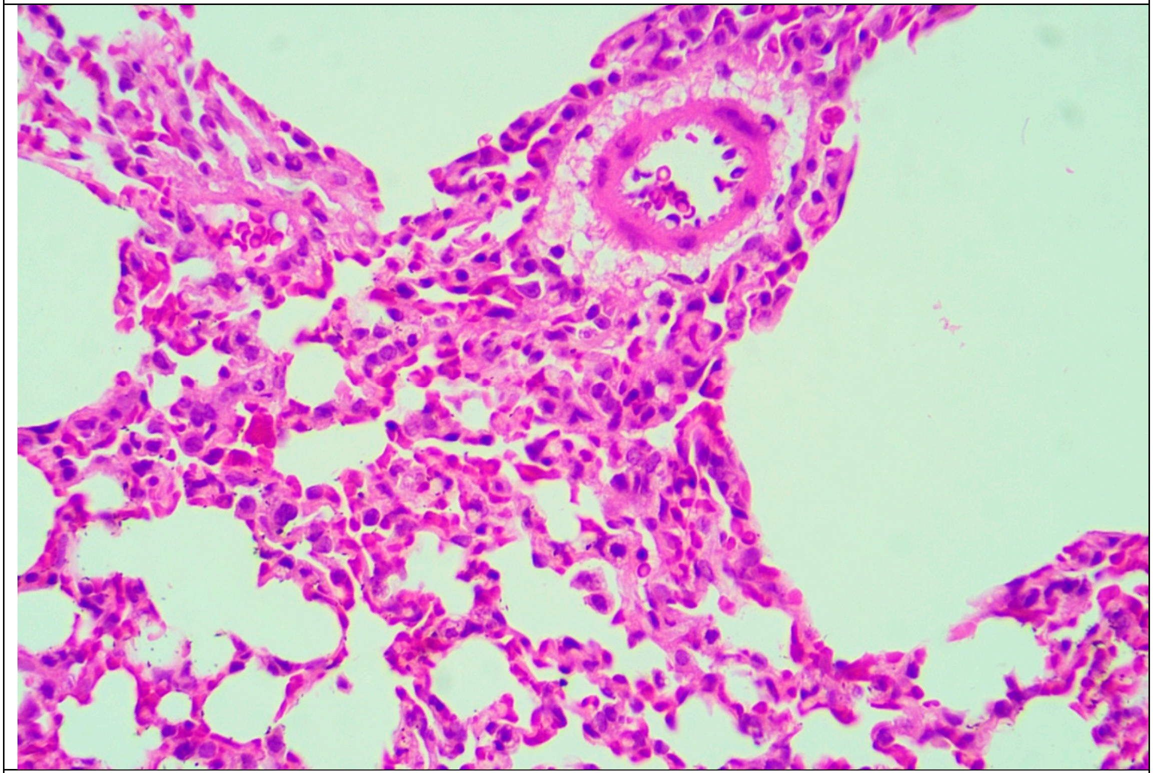

D

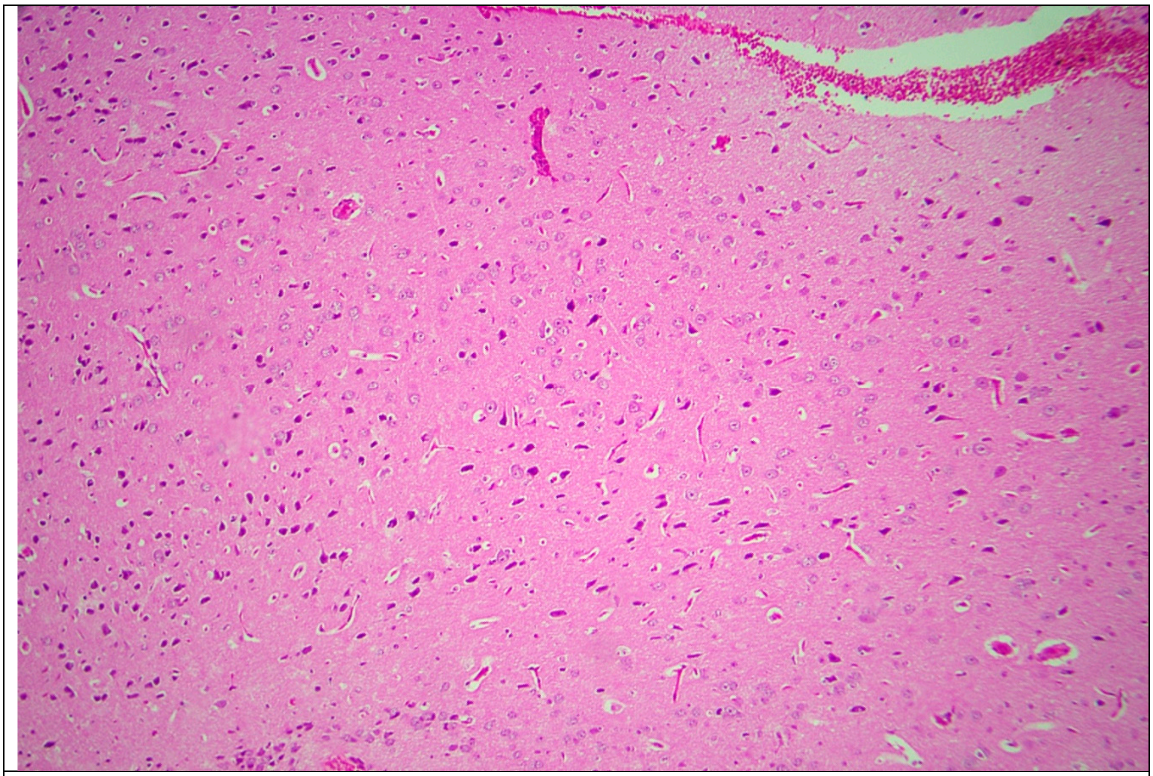

E

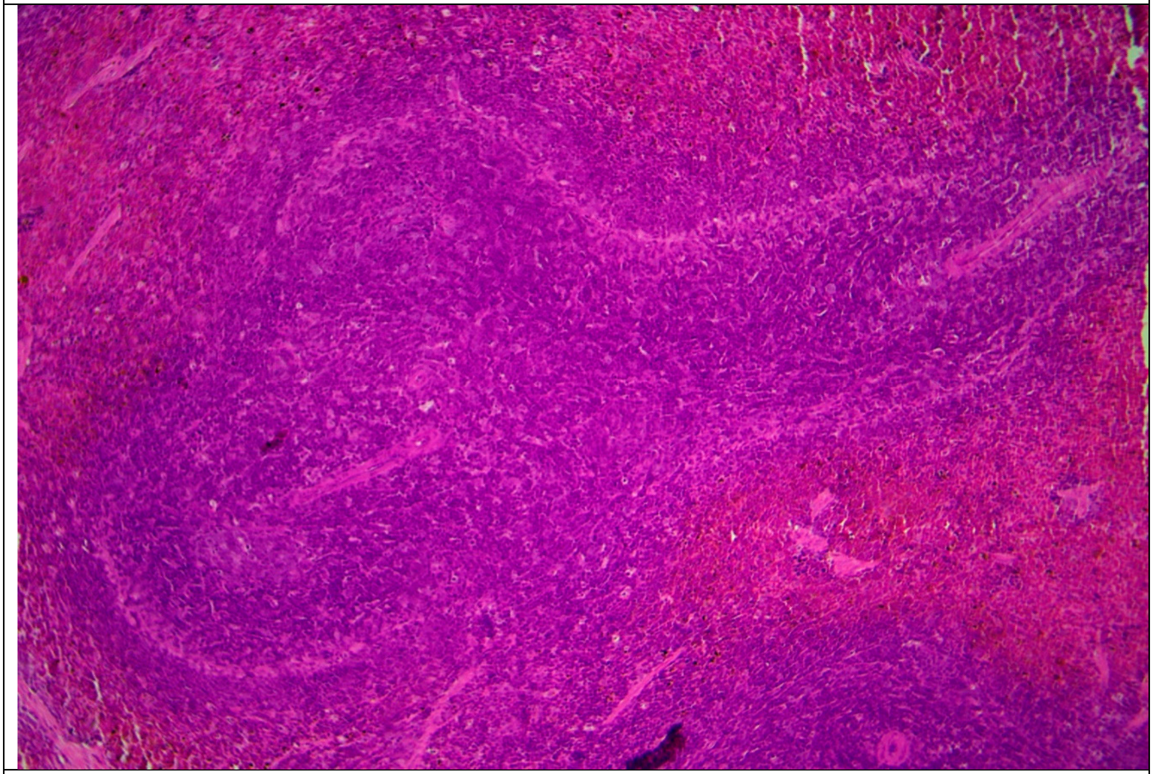

F

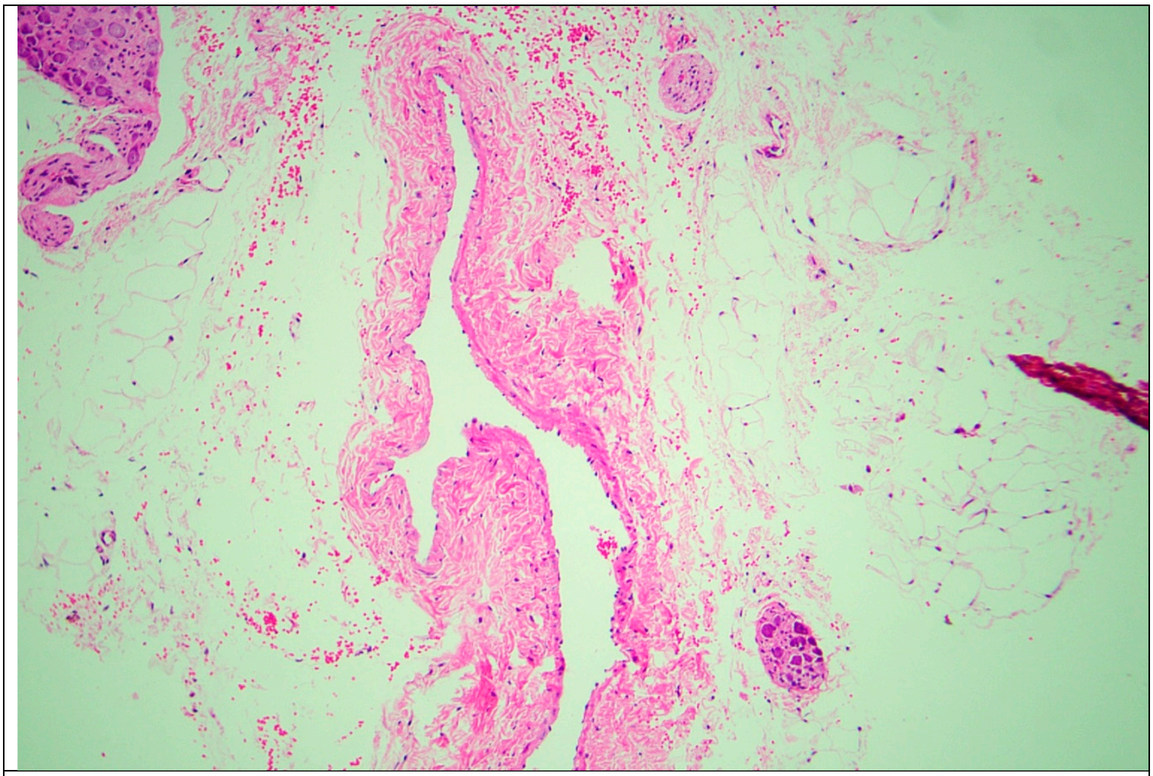

G

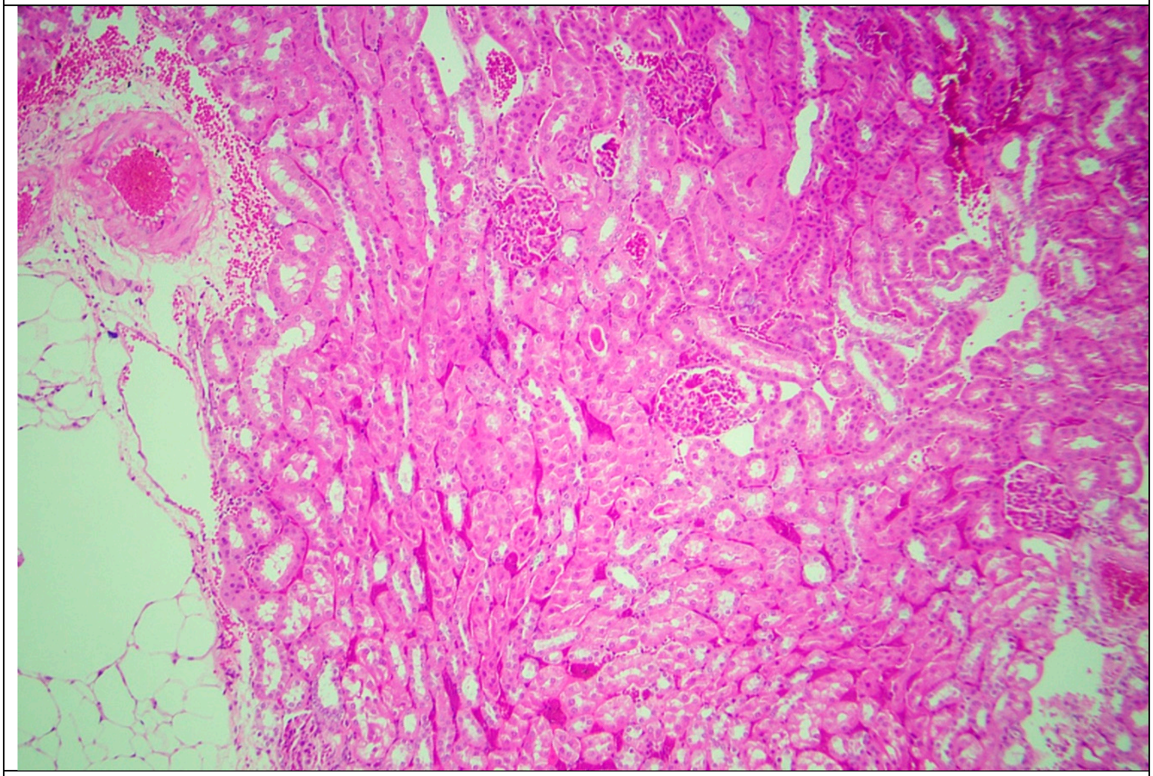

H

Figure S1. Histopathological analysis of different hamster tissues. A and B correspondent to lesions of a genetic background normally found in the kidney and liver. (A) lesions in the kidney: focal membranoproliferative glomerulopathy and moderate multifocal lymphocytosis in the interstitial tubule. (B) mild multifocal periportal plasmacytosis in the liver. C to H correspondent to histopathological analysis animal control tissues.
