# Supplementary material for: Assessment of the Pathogenicity of Candidatus Rickettsia Colombiensis in a Syrian Hamster Model and Serological Cross-Reactivity Between Spotted Fever Rickettsia Species
Source: Pathogens. 2026 Jan 29;15(2):146. doi: 10.3390/pathogens15020146 (PMC12943140; doi:10.3390/pathogens15020146)
Supplement: Supplementary file 1 [file pathogens-15-00146-s001.zip › Table S1.pdf]

Table S1. *Rickettsia* species and the GenBank accession number used to construct the phylogenetic tree of partial *ompA* gene.

	**Especie**	**Country**	**GenBank accession**
1	Rickettsia cepa Adcor5	Colombia	PQ658084
2	*Candidatus* Rickettsia colombianensi	Brasil	MG970683
3	Rickettsia sp. strain colombianensi	Colombia	JF905458
4	*Rickettsia amblyommatis*	Brasil	MF188911
5	*Rickettsia honei*	Australia	AF018075
6	*Rickettsia slovaca*	France	U43808
7	*Rickettsia africae*	Ethiopia	U43790
8	*Rickettsia parkeri*	USA	U43802
9	*Rickettsia sibirica*	Rusia	U43807
10	*Rickettsia conorii*	India	U43794
11	*Rickettsia japonica*	Japon	U43795
12	*Rickettsia massiliae*	France	U43799
13	*Rickettsia rhipicephali*	USA	U43803
14	*Rickettsia monacensis*	Czech Republic	MN853331
15	*Rickettsia australis*	Australia	AF149108
16	*Rickettsia peacockii*	USA	AY319292
17	*Rickettsia montanensis*	USA	U43801
18	*Rickettsia rickettsii*	USA	U43804
19	*Rickettsia conorii*	South Africa	U43806
20	*Rickettsia mongolotimonae*	Mongolia	U43796
21	*Rickettsia tamurae*	Japon	DQ103259
22	*Rickettsia aeschlimannii*	Morroco	U43800
